# Early increase in circulating carbonic anhydrase IX: A potential new predictive biomarker of preeclampsia

**DOI:** 10.3389/fmolb.2023.1075604

**Published:** 2023-01-19

**Authors:** Silvia Galbiati, Daniela Gabellini, Alessandro Ambrosi, Nadia Soriani, Federica Pasi, Massimo Locatelli, Roberta Lucianò, Massimo Candiani, Luca Valsecchi, Gianpaolo Zerbini, Maddalena Smid

**Affiliations:** ^1^ Complications of Diabetes Unit, Diabetes Research Institute, IRCCS San Raffaele Scientific Institute, Milan, Italy; ^2^ School of Medicine and Surgery, Vita-Salute San Raffaele University, Milan, Italy; ^3^ Unit of Genomic for the Diagnosis of Human Pathologies, Division of Genetics and Cell Biology, IRCCS San Raffaele Scientific Institute, Milan, Italy; ^4^ Obstetrics and Gynecology Department, IRCCS San Raffaele Scientific Institute, Milan, Italy; ^5^ Laboratory Medicine Service, IRCCS San Raffaele Hospital, Milan, Italy; ^6^ Department of Pathology, IRCCS San Raffaele Hospital, Milan, Italy

**Keywords:** CAIX, biomarker, hypoxia, pregnancy, preeclampsia

## Abstract

Preeclampsia (PE) is a severe complication of pregnancy. The identification of a reliable predictive biomarker could help in setting up a specific preventive strategy. To this aim, we studied carbonic anhydrase IX (CAIX) as a marker of hypoxia (a pathway involved in PE pathogenesis) and compared the diagnostic accuracy of CAIX to that of the validated biomarker sFlt1/PlGF ratio. Fifteen women with overt PE and 38 women at a risk of developing PE, sampled at different time intervals during gestation (a total of 82 plasma samples collected), were enrolled and underwent the CAIX measurement. CAIX levels significantly increased (*p* < .001) before the onset of the disease in women (25% of the total number) who later on developed PE when compared to women who did not, starting from 28th gestational week. The best CAIX cut-off of 68.268 pg/mL yielded a sensitivity of 100%, a specificity of 81.82%, and an AUC value of .9221. In our pilot study, when compared to the sFlt1/PlGF ratio, CAIX performed better in predicting PE before the clinical onset. Furthermore when implemented as CAIX/PlGF ratio, showed up to be comparable in the identification of women with overt early PE. In conclusion, CAIX could represent an effective predictive biomarker of PE, and larger studies are mandatory to validate this finding.

## 1 Introduction

Preeclampsia (PE) is a hypertensive pregnancy condition that affects approximately 3%–5% of women in the second half of pregnancy, causing a significant fetal/perinatal and maternal mortality and morbidity worldwide, especially in low- and middle-development countries (Chappell et al., 2021).

PE presents clinically as at least two different disorders: an early-onset form that occurs before 34 weeks of gestation and a late-onset form that occurs after 34 weeks of gestation. The early onset form is more severe despite being less frequent and associated with abnormal placentation and intra-uterine growth restriction, while the late-onset form is often related to maternal metabolic disorders (Burton et al., 2019; Roberts et al., 2021). Pathogenic alterations differentially involved in early and late PE onset are mainly placental underperfusion, endothelial damage, oxidative stress and hypoxia, inflammation, maternal constitutional disorders, and a genetic background. Our ability to identify pregnant women who are at the highest risk of developing the pathology is fundamental for cost-effective distribution of monitoring resources and the use of preventive treatments. In recent years, lots of studies have concentrated their efforts on identifying predictive biomarkers in order to detect the risk of developing PE before the clinical onset. Several studies focused on the imbalance between pro-angiogenic factors (such as the vascular endothelial growth factor (VEGF) or placental growth factor (PlGF)) and anti-angiogenic factors such as soluble fms-like tyrosine kinase 1 (sFlt1) (Stepan et al., 2007; Lim et al., 2008). sFlt1 is a circulating decoy receptor of VEGF. Even if in a limited way, an increased sFlt1 plasma concentration and, consequently, VEGF inhibition also occur in physiological pregnancy (Buhimschi et al., 2006), but in this particular case, dysfunction is counterbalanced by the simultaneous increased secretion of PlGF by the placenta. Circulating levels of sFlt1 and PlGF are abnormal in PE patients. This variation begins before the onset of the disease and remains throughout the course of the disease. In women who develop PE, sFlt1 increases almost 5 weeks before the disease onset, while the level of PlGF decreases before the rising of sFlt1 (Nikuei et al., 2020). In particular, it is established that a sFlt1/PlGF plasma ratio of 38 or lower can be used to predict the short-term absence of pathology within 7 days in women in whom the syndrome is clinically suspected (a negative predictive value of 99.3%) (Rana et al., 2022). Conversely, a ratio >38 conferred a positive predictive value (PPV) of 36.7% (95% CI, 28.4–45.7) that poorly correlated with the development of preeclampsia within 4 weeks (Cerdeira et al., 2019). In overt preeclampsia, the ratio has a high sensitivity (99.5% or 95.5% in women before the 34th gestational week or after, respectively). The values of the sFlt1/PlGF plasma ratio >85 before 34 weeks or >110 after 34 weeks of gestation are those recommended in the current clinical practice as alert values (Baert et al., 2021). In our previous study, we explored longitudinally throughout gestation some possible predictive mRNA biomarkers of PE in a high-risk population, involving different pathogenic pathways including endothelial damage. Among these pathways, we also explored hypoxia. We observed that the expression of hypoxia-inducible factor-1A (HIF1A), a strategic regulator of cellular metabolism and the response to hypoxic conditioning, was the earliest marker associated with the PE development (Galbiati et al., 2015). In particular, we observed that until 23 weeks of gestation, HIF1A was significantly higher in women who later on developed PE than women who did not (Galbiati et al., 2015). In this study, to further investigate hypoxia, we looked at carbonic anhydrase IX (CAIX), a downstream target of HIF1A. CAIX is a glycoprotein that supports angiogenesis, cell proliferation, and cell survival under hypoxic environments through its role in pH regulation. Although CAIX is a transmembrane protein, hypoxia induces shedding of its extracellular domain, which can be released into body fluids (Irvine et al., 2020). In pregnancy, CAIX is hypothesized to be a marker of placental hypoxia (Ravishankar et al., 2015), placental dysfunction, and poor pregnancy outcomes including fetal growth restriction (Brown et al., 2018). Increased levels of CAIX in plasma have also been identified in women with overt preeclampsia (Valsecchi et al., 2022) or with hemolysis, elevated liver enzymes, and low platelet count (HELLP) syndrome, which is a complication of severe preeclampsia (Mentese et al., 2018). Plasma CAIX levels have never been used to predict PE. The aim of this study was, first, to study longitudinally the expression of CAIX in women at risk of developing PE. The second aim was to evaluate the diagnostic accuracy of CAIX comparing the results with those obtained using the sFlt1/PlGF ratio not only in women with overt PE but also in women at risk of developing the disease before the onset of the pathology.

## 2 Materials and methods

### 2.1 Patients

For the analysis, 6 mL of peripheral blood samples from pregnant Caucasian women, followed by the Obstetric and Gynecology Unit of the San Raffaele Scientific Institute of Milan, were collected in vacutainer tubes containing EDTA. All the pregnant women enrolled in this study provided written informed consent. This study was approved by the local Ethical Review Boards. Two groups of women were considered: i) 15 women with overt PE enrolled at the time of diagnosis (24–38 weeks), among which 8 developed early- onset PE (ranging from 26th to 34th week, median 29 weeks), and 7 developed late-onset PE (>34 weeks of gestation, median 36 weeks); ii) women with a familial history of PE having a risk of developing PE, history of previous pregnancy complication by PE, or with chronic hypertension sampled consecutively at 16–18 (*n* = 23 and *n* = 7, women developed PE); 20–24 (*n* = 26 and *n* = 6, women developed PE); 28–32 (*n* = 29 and *n* = 7, women developed PE), and 34–36 (*n* = 4, no woman developed PE) gestational weeks. The results of the analysis concerning the samples of women at risk collected at the time of diagnosis, if available, were included in the correspondent group of the overt pathology (*n* = 3). According to ACOG guidelines, PE was defined as elevated systolic blood pressure (≥140 mmHg) or diastolic (≥90 mmHg) on at least two different occasions in a woman who was normotensive before 20 weeks of gestation and persistent proteinuria (≥300 mg/24 h).

### 2.2 Plasma separation

Plasma was separated from the total blood by two centrifugation steps (4° C) of 10 min each, the first at 1,600 g and the second one at 14,000 g, and stored at −80° C until further processing analysis*.*

### 2.3 Assessment of CAIX

Concentrations of CAIX in plasma were measured using an enzyme-linked immunosorbent assay (ELISA) kit, namely, the Quantikine^®^ ELISA Human CAIX Immunoassay DCA900 kit (R&D Systems, Minneapolis, MN, United States), according to the manufacturer’s instructions. The absorbance of samples was measured at 450 nm, using a microplate reader (Bio-Rad Model 680). The results were expressed as pg/ml. The calibration curve ranges from 15.6 to 1,000 pg/ml. At low values, the coefficient of variability is 3.8% (intra-assay) and 6.3% (inter-assay) (Valsecchi et al., 2022). In case of low values (below the limit of detection), the data were censored and substituted with a constant value, equal to half the limit of detection (7.8 pg/ml) (Wood et al., 2011).

### 2.4 Assessment of sFlt1, PlGF and sFlt1/PlGF ratio

Plasma levels of sFlt1 and PlGF were assessed using the Roche Elecsys^®^ sFlt-1 and Elecsys^®^ PlGF assays on the electrochemiluminescence immunoassay platform Cobas^®^ 6000 (Roche Diagnostics GmbH, Mannheim, Germany). The sFlt1/PlGF ratio cut-off values with ≥85 for ≤34 weeks of gestation and ≥110 for >34 weeks of gestation were used to categorize the women as overt PE.

### 2.5 Statistical analysis

Continuous variables were summarized by mean values and standard deviation (sd) values. Categorical variables were summarized by frequencies and percentage. The distribution of the observed valued was graphically represented by the boxplot and heatmap. For each biomarker, the overall performance in distinguishing couple of groups was measured by the area under the curve (AUC) of the associated ROC curve. Confidence intervals for ROC curves were computed by the bootstrap method (B = 5,000). Optimal cut off were identified based on the Youden criterion maximizing Youden’s J statistic (sensitivities + specificities). The relationship between the different biomarker was further investigated by linear regression. To further investigate the pattern of CAIX as a function of time, we fitted a cubic spline function for each group and plotted them with the associated confidence interval.

The probability of PE was estimated by the GLM model, considering as independent variables the values of the biomarker with respect to the relative optimal threshold. *p*-values less than .05 were considered significant. All the analyses were computed in an R 4.1.3 environment.

## 3 Results

### 3.1 Clinical features of study samples

Demographic maternal characteristics concerning age, body mass index, gravity and parity, ethnicity, and smoking habits did not significantly differ between the women at risk who were subsequently diagnosed as PE and those who did not, as reported in Table 1. On the contrary, unsurprisingly, gestational week at delivery and birth weight were lower in women at risk who developed PE.

**TABLE 1:** Anamnestic data on our population of women at risk to develop PE.

	Women at risk who did not develop PE	Women at risk who developed PE	*p*-value
Maternal age (mean, years)	34.63	38.18	.1027
BMI (mean, kg/m^2^)	26.28	25.27	.9709
Ethnicity (Caucasian)	100%	100%	-
Smoking habits (median)	0	0	-
Gravity (mean)	2.074	1.727	.2022
Parity (mean)	.4074	.1818	.1963
Neonatal birth weight (mean, g)	3,256	2,445	.0058
Gestational age at delivery (mean, weeks)	38.56	34.4	< .0001

### 3.2 CAIX plasma concentrations in the study population

**FIGURE 1:**
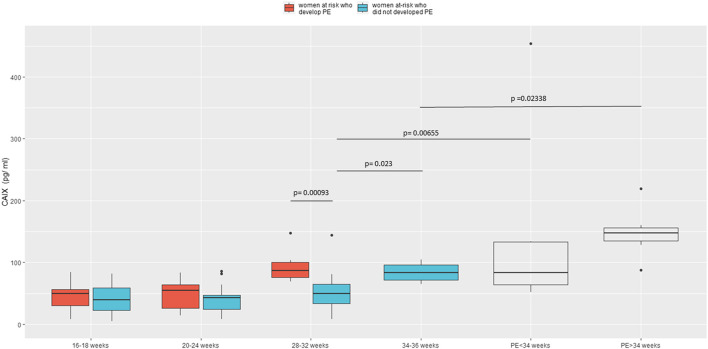
Box plots comparing CAIX levels in women at risk who were subsequently diagnosed as PE (red) and those who were not (light blue) sampled at different gestational ages and women with the early (*n* = 8) or late (*n* = 7) overt PE (white). 16–18 gestational weeks: *n* = 7 women at risk who developed PE and *n* = 16 women at risk who did not develop PE; 20–24 gestational weeks: *n* = 6 women at risk who developed PE and *n* = 20 women at risk who did not develop PE; 28–32 gestational weeks: *n* = 7 women at risk who developed PE and *n* = 22 women at risk who did not develop PE; 34–36 gestational weeks: *n* = 4 women at risk who did not develop PE.

In our longitudinal assessment of women at risk of developing PE, we compared CAIX expression levels in women who subsequently developed the disease *versus* women who did not at four different progressive intervals: between 16–18, 20–24, 28–32, and 34–36 gestational weeks. As shown in Figure 1, CAIX expression significantly increased (*p* = .023) in women at risk who did not develop PE through gestation. Starting from 16 until 24 weeks, CAIX was similarly expressed in women who later on developed the pathology compared to women who did not. However, starting from the 28th gestational week, CAIX significantly increased before the onset of the disease (*p* = .00093) in women who later on developed the pathology (within 2–7 weeks) compared to women who did not (Figure 1). We also found a significant difference in CAIX expression (*p* = .00655) among women with early overt PE (<34th gestational week) compared to women at risk who did not develop PE matched for gestational age (28–32 gestational weeks). A significant increase of CAIX (*p* = .02338) was also observed in women with late overt PE (>34th gestational week) *versus* women at risk who did not develop PE at a later gestational age (34–36 weeks) (Figure 1).

**FIGURE 2:**
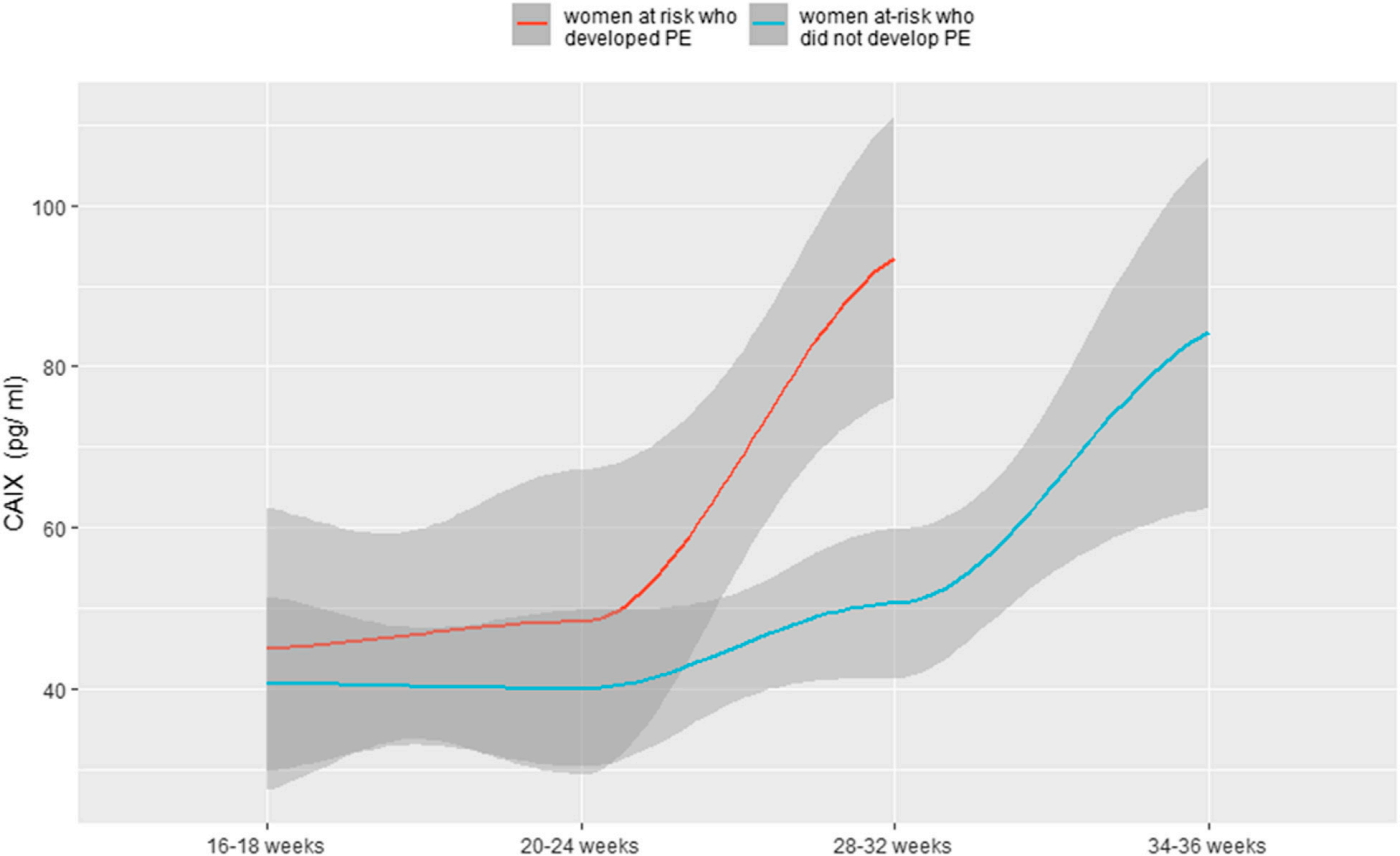
Levels of CAIX as a function of gestational time for both women at risk who did not develop PE and women at risk who developed PE.

To further investigate the pattern of CAIX between women who developed preeclampsia and those who did not as a function of time, we fitted a cubic spline function for each group and plotted them with the associated confidence interval (Figure 2).

**FIGURE 3:**
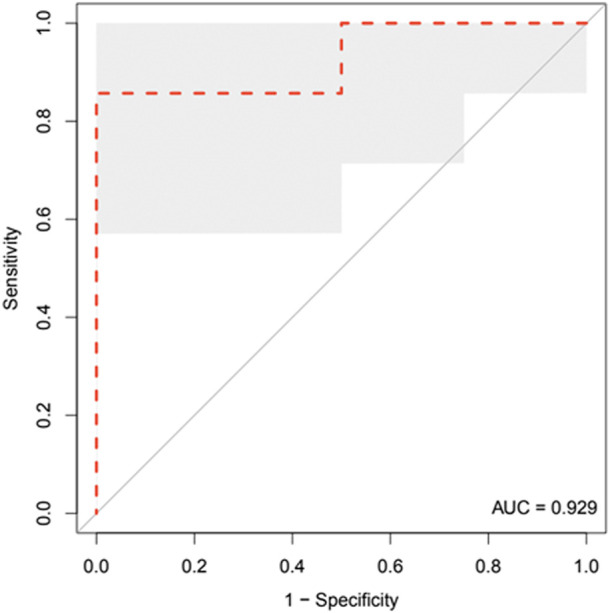
ROC curve analysis of CAIX in women who later developed the pathology (*n* = 7) compared to women who did not (*n* = 22) at a gestational age ranging from 28 to 32 gestational weeks. The shadowed area represents the bootstrap 95% confidence interval.

In particular, as shown in the ROC curve of Figure 3 [AUC = .9221: 95%CI: (80.52, 100)], CAIX assay at 28–32 gestational weeks based on the best cut-off, according to Youden’s J statistic, of 68.268 pg/ml yielded a sensitivity of 100% and a specificity of 81.82%. The positive predictive value (PPV) and negative predictive value (NPV) were 64% and 100%, respectively, with an overall diagnostic accuracy of 86%.

### 3.3 Comparative analysis of CAIX *versus* the sFlt1/PlGF ratio in clinical practice

A subgroup of 60 women analyzed for the CAIX biomarker was also studied for sFlt1 and PlGF alone or combined (ratio) to evaluate the diagnostic accuracy of CAIX, comparing the results to those obtained using the ratio. In particular, we analyzed: i) five women with overt early PE collected at the time of diagnosis (<34 weeks of gestation); ii) women at risk of developing PE sampled consecutively at 16–18 (*n* = 18 and *n* = 7 women developed PE), 20–24 (*n* = 18 and *n* = 6 women developed PE), and 28–32 (*n* = 19 and *n* = 7 women developed PE) gestational weeks. None of the biomarkers studied alone or even combined were differently expressed among women at risk who later on developed the pathology compared to women who did not until 24 gestational weeks, while, as expected, CAIX was significantly higher (*p* = .00073) before the onset of the disease in women who later on developed the pathology compared to women who did not, starting from the 28th week. A similar but less significant result was obtained using sFlt1 alone (*p* = .01800), while using PlGF alone or even combined with sFlt1 (ratio) statistical significance was not achieved. In Table 2, the values of circulating levels of CAIX, sFLt1, and PlGF in the third time window (28–32 gestational weeks) were reported.

**TABLE 2:** Value of circulating CAIX, sFlt1, and PlGF for individual women.

Women at risk who did not develop PE (n = 12) (28–32 gestational weeks)	CAIX (pg/ml)	sFlt1 (pg/ml)	PlGF (pg/ml)
53MI	58,063	1,471	357
90MI	39,859	1991	1,035
97MI	37,799	1,261	1,330
63MI	7,8	993	526
69MI	48,807	1,189	282
70MI	36,39	1,275	362
71MI	7,8	926	364
80MI	50,012	1,331	216
47MI	26,316	1,283	231
43MI	32,726	1,420	1,153
32MI	37,122	967	125
27MI	74,316	5,051	123
Mean	38,08	1,597	508,7
Standard deviation	19,02	1,124	420,2

Women at risk who developed PE (*n* = 7) (28–32 gestational weeks)	**CAIX (pg/ml)**	**sFlt1 (pg/ml)**	**PlGF (pg/ml)**
67MI_late	97,151	4,582	161
60MI_early	147,202	11,245	132
39MI_late	69,174	2,117	613
41MI_early	68,666	1,008	1,509
77MI_late	103,589	2085	207
3MI_late	86,915	2,690	244
8MI_late	82,168	4,126	90
Mean	93,55	3,979	422,3
Standard deviation	27,02	3,433	509,6

**FIGURE 4:**
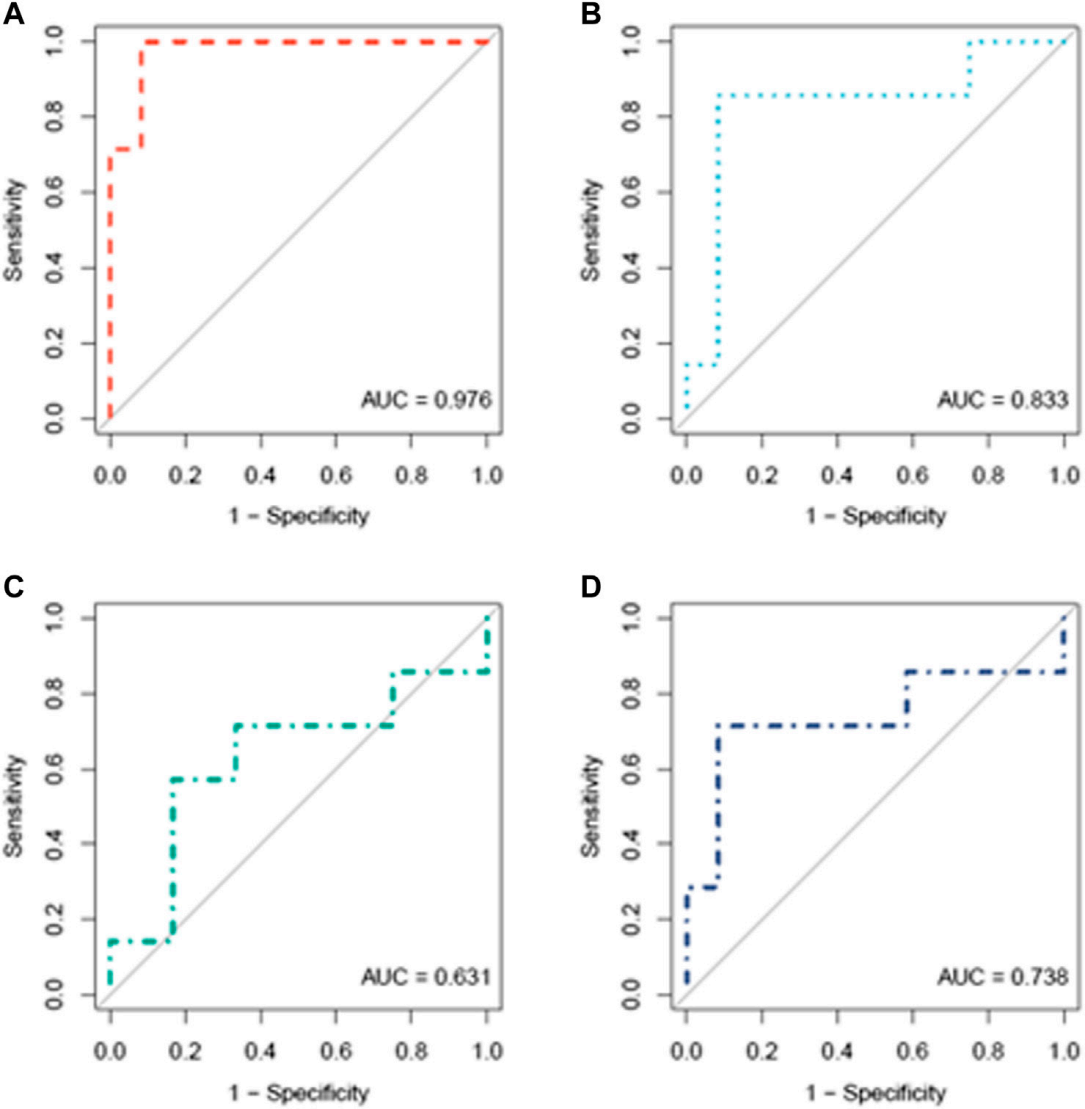
ROC curve analysis of CAIX **(A)**, sFlt1 **(B)**, PlGF **(C)**, and sFlt1/PlGF **(D)** in women who later developed the pathology (*n* = 7) compared to women who did not (*n* = 12) at a gestational age ranging from 28 to 32 weeks.

In Figure 4, the ROC curves of CAIX, sFlt1, and PlGF alone or combined with sFlt1 are shown. In Table 3, for each biomarker, specificity, sensitivity, accuracy, and negative and positive predictive values are reported based on the best cut-off chosen.

**TABLE 3:** Specificity, sensitivity, accuracy, negative predictive value (NPV), and positive predictive value (PPV) of the different biomarkers.

Biomarker	Cut-off (pg/ml)	Specificity	Sensitivity	Accuracy	NPV	PPV
CAIX	63.364	.916	1.00	.947	1.00	.875
sFlt1	2038	.916	.857	.894	.917	.857
PlGF	211.5	.833	.571	.736	.769	.666
sFlt1/PlGF	8.904	.916	.714	.842	.846	.833

**FIGURE 5:**
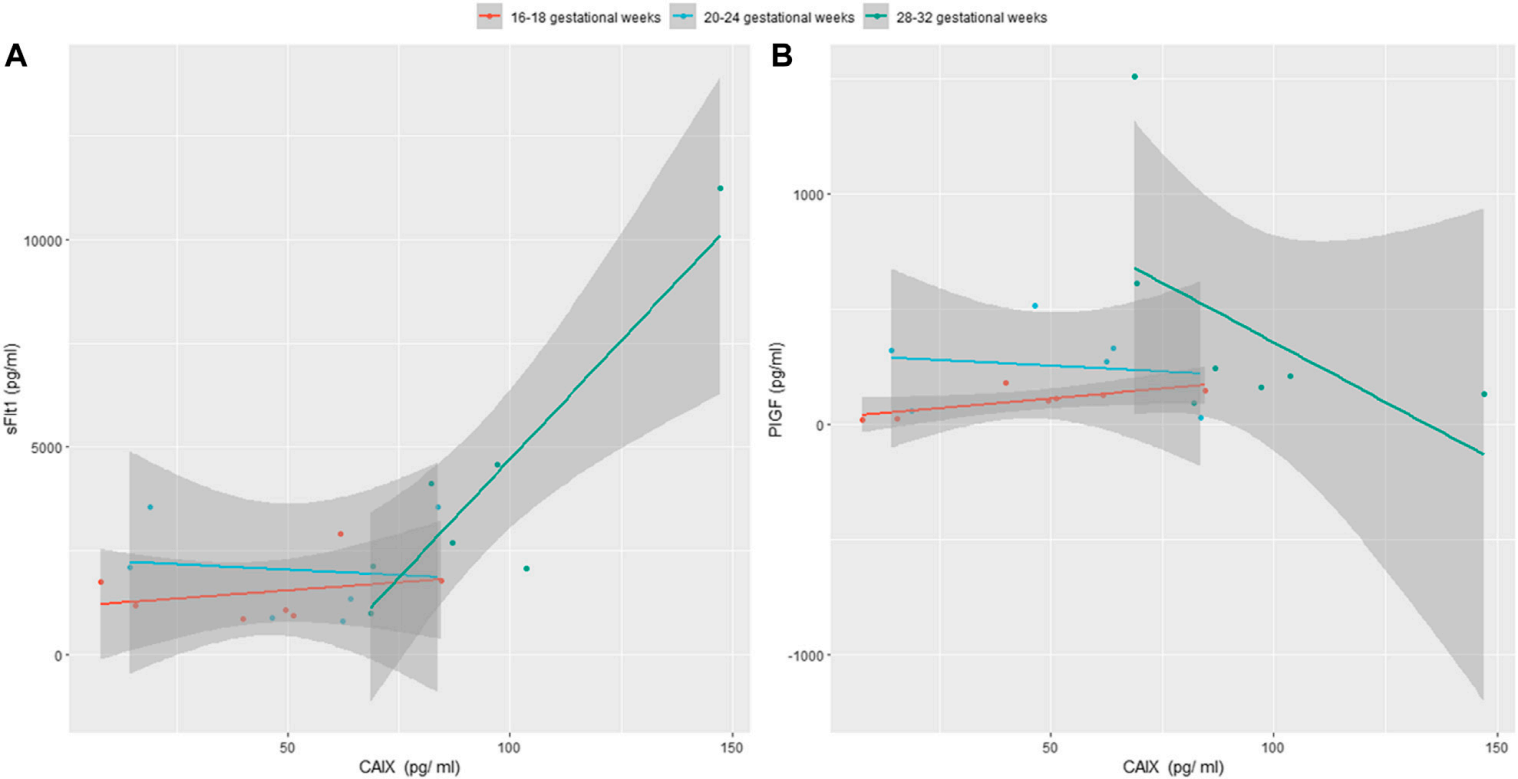
**(A)** Positive correlation among CAIX and sFlt1 in women who will develop PE sampled consecutively at 16–18, 20–24, and 28–32 gestational weeks is shown; **(B)** negative correlation between CAIX and PlGF in the same cohort of women is shown.

**FIGURE 6:**
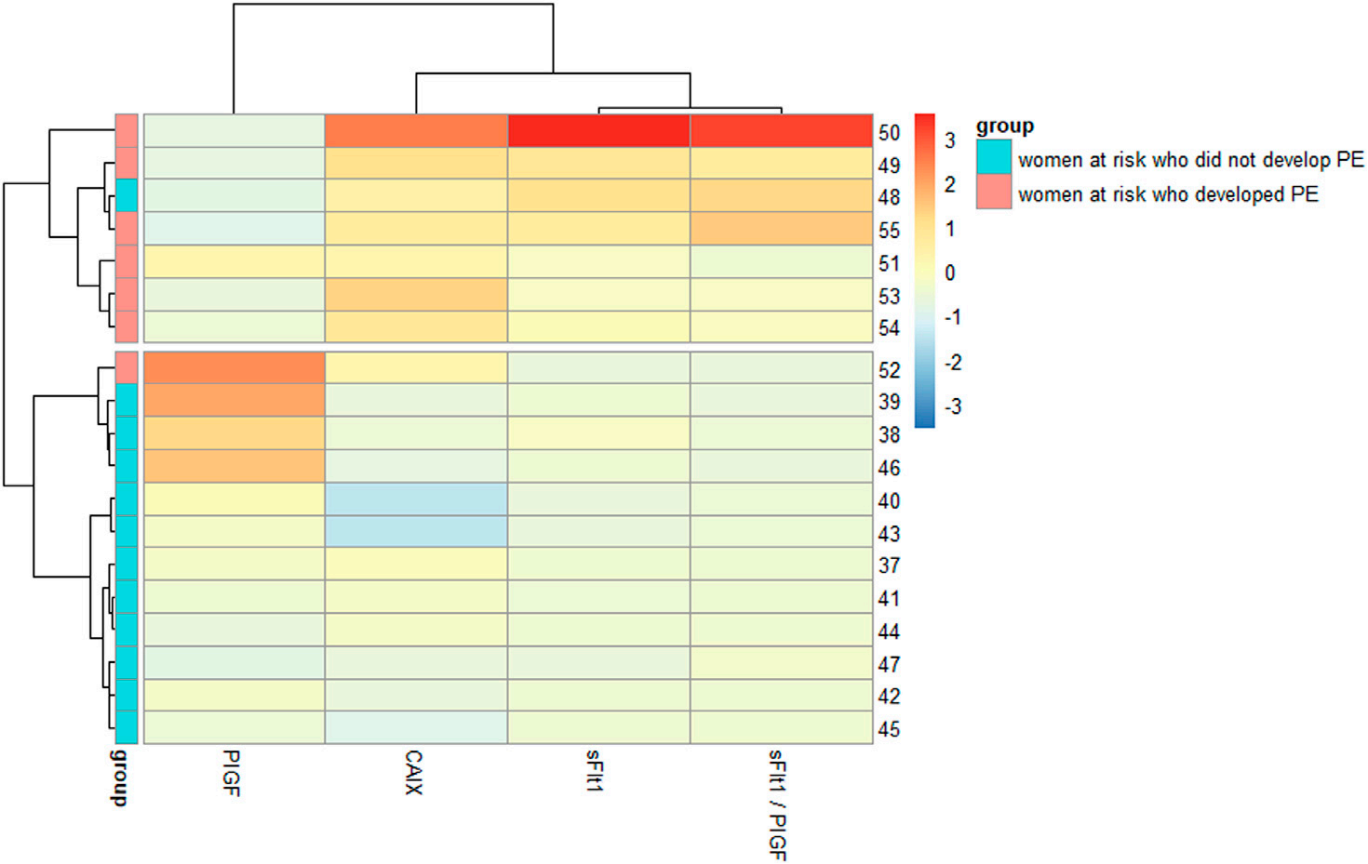
Heatmap of CAIX, sFlt1, and PlGF in women who later developed the pathology and in women who did not at 28–32 gestational weeks.

**FIGURE 7:**
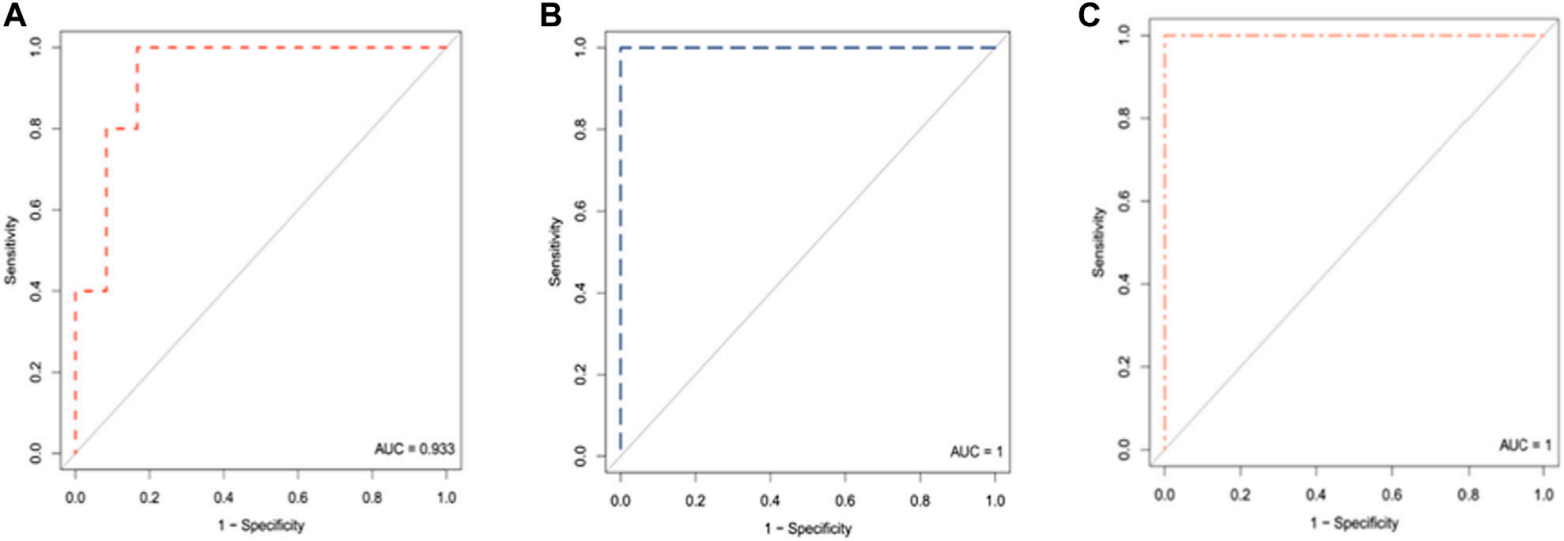
ROC curve analysis of CAIX **(A)**, sFlt1/PlGF ratio **(B)**, and CAIX/PlGF ratio **(C)** in women who did not develop the pathology (*n* = 12) at a gestational age ranging from 28 to 32weeks compared to women with early overt PE (*n* = 5).

Interestingly, we noted that CAIX levels were positively correlated with sFlt1 both increasing during gestation (28–32 gestational weeks) in women who developed PE (*p*-value = .006184 and correlation coefficient = .897) and inversely correlated with PlGF (*p*-value = .2013 and correlation coefficient = −.549), as shown in Figure 5. The heatmap of Figure 6 visualizes the features of the three different biomarkers (alone or combined) in women who later on developed the pathology (*n* = 7) compared to women who did not (*n* = 12) at a gestational age ranging from 28 to 32 weeks. The change in intensity across the different women in the two cohorts displayed *via* a line graph showed that individual biomarkers behave differently between the two cohorts with a best overall performance of the biomarker CAIX. However, if we combine CAIX levels (which even alone is associated with a very high risk of developing PE) with both sFlt1 and PlGF levels or even with PlGF alone, we obtain a very useful predictor tool that allow us in identifying women with a critical risk for developing PE when sampled between 28 and 32 gestational week, as reported in Table 4. It is well established that the best clinical performance of the sFlt1/PlGF ratio is not related to the early prediction of the development of PE but to the capacity to identify overt PE or to rule out women at risk with suspected signs of preeclampsia for the following week. Thus, in conclusion, we compared CAIX, sFlt1/PlGF, and even CAIX/PlGF levels (due to the positive correlation of CAIX with sFlt1 shown in Figure 5) from women at risk who did not develop PE (median 29.5 gestational week) to women with early overt PE (median 28 gestational week) sampled at the same gestational age. The corresponding ROC curves are shown in Figure 7. CAIX, even if it could be considered a useful biomarker also in overt PE diagnosis, it performs worse than the sFlt1/PlGF ratio. However, when implemented as the CAIX/PlGF ratio, it showed up to be comparable in the identification of women with overt early PE.

**TABLE 4:** Fitted probability of PE as a function of the expression of the different biomarkers. “Low” and “high” values are referred to the relative optimal cut-offs reported in Table 3. Given the sample size, the values should be considered carefully.

CAIX	sFlt1	PlGF	sFlt1/PlGF	Estimated probability	Risk class
**Low**	High	Low	High	<.00001	Low
Low	Low	Low	High	<.00001	Low
Low	High	Low	Low	<.00001	Low
Low	Low	Low	Low	<.00001	Low
Low	Low	High	High	<.00001	Low
Low	High	High	High	<.00001	Low
Low	Low	High	Low	<.00001	Low
Low	High	High	Low	<.00001	Low
High	Low	Low	High	0.8	High
High	High	Low	High	0.8	High
High	Low	Low	Low	0.9	Very high
High	High	Low	Low	0.9	Very high
High	High	High	High	1	Critical
High	Low	High	High	1	Critical
High	High	High	Low	1	Critical
High	Low	High	Low	1	Critical

## 4 Discussion

At the moment, the pathogenesis of PE is not fully defined. However, the imbalance between angiogenic factors like VEGF or PlGF and anti-angiogenic factors like sFlt1 released in the circulation by the placenta is known to be involved in the disease development (Baert et al., 2021; Verlohren et al., 2022). Thus, these markers, alone or combined, could be used for the management of suspected preeclampsia. In particular, latest studies showed that the sFlt1/PlGF ratio due to its high negative predictive value is a reliable exclusion test for the development of preeclampsia within 7 days, reducing unnecessary medical intervention (Cerdeira et al., 2019). Unfortunately, on the contrary, its positive predictive value remains poor, limiting the use of the ratio as a diagnostic tool (Cerdeira et al., 2019). Many research groups are still actively searching for early predictive biomarkers, especially in women with a higher risk to develop the pathology, looking for different pathways that could explain the involvement of multiple organ systems, as in PE (Ives et al., 2020; Rasmussen et al., 2022). The inability of the trophoblasts to reach uterine arteries in women who will develop PE generates hypoxia-induced oxidative stress (Hu and Zhang, 2021) and imbalance of angiogenic factors. Upregulation of HIF1A has been reported in the PE pathogenesis as a main regulator of the cells in response to low oxygen levels (Tianthong and Phupong, 2021; Valsecchi et al., 2022). In normoxic conditions, HIF1A is inactivated and degraded; however, in hypoxic conditions, HIF1A regulates the expression of several genes (among which VEGF and CAIX) by binding to the hypoxia-responsive element at the 5′ end to their transcriptional start site with consequent hypoxic upregulation of these genes (Tianthong and Phupong, 2021). In this study, we have investigated the relationship between the expression of CAIX and preeclampsia. We observed that CAIX levels increase during pregnancy (especially at the end of gestation) in women at risk of developing PE who eventually have uncomplicated pregnancies. However, CAIX levels rise more consistently in pathological pregnancies. CAIX was significantly increased (*p* < .001) in women at risk who later on (within 2–7 weeks) developed the disease starting from the 28th gestational week. These data are consistent with those that have been already described by our and other groups, suggesting hypoxia may play an important role in PE pathogenesis (Galbiati et al., 2015; Brandão Tenório et al., 2019; Tianthong and Phupong, 2021). Indeed, in our previous study, increased HIF1A mRNA was the earliest marker found in association with the PE development even before the 28th gestational week (Galbiati et al., 2015). When we compared the data obtained by CAIX analysis with those obtained by sFlt1 and PlGF alone or combined, we observed that sFlt1 alone was less sensitive than CAIX (as reported in Table 3) in discriminating women at risk who later developed PE *versus* women at risk who were not sampled between 28th and 32nd weeks. Moreover, PlGF lacked any discriminating activity in our cohort of women sampled in the second and third trimesters (Bujold 2022). Thus, CAIX could be considered a suitable predictive biomarker in women at risk for PE, helping the clinicians to assess the mother and fetuses who need a close surveillance. On the contrary, CAIX levels <63.364 pg/ml are highly reassuring and should encourage minimal medical intervention. As reported in Table 4, high levels of CAIX alone allow defining “high-risk” *versus* “low-risk” pregnant women of developing PE. However, an improvement in the classification at “critical risk” to develop the pathology could be achieved analyzing in parallel to at least PlGF. This could probably be due to the inverse correlation shown between CAIX and PlGF that could sum the predictive effects of the two biomarkers. CAIX analysis seems to be useful not only as the predictive biomarker of PE but also as a diagnostic biomarker identifying women with early or late overt PE. Concerning the source of CAIX found in the plasma of pregnant women, there are some pieces of evidence that CAIX is expressed in the villous cytotrophoblast in early pregnancy and in the chorionic plate mesenchymal cells during all gestation periods (Liao et al., 2009). The expression of CAIX, as a biomarker of hypoxia in pregnancy, was evaluated by Ravishankar et al., in physiological placentas and in placentas of women with obstructive sleep apnea (OSA), showing positive membranous staining in the chorionic plate mesenchyme layer and weak cytoplasmic staining in amniotic epithelium (Ravishankar et al., 2015). In addition, only in the placenta of women with OSA, positive membranous staining in extravillous trophoblast cells at the basal plate was also observed (Ravishankar et al., 2015). These cells are involved in the physiologic alteration of the decidual vessels, and their differentiation and function are influenced by oxygen tension. Changes in these processes appear to be the cause of pregnancy disorders, including PE (Lunghi et al., 2007). Whether the source of CAIX is fetal or maternal remains to be clarified (Ravishankar et al., 2015). Furthermore, we cannot exclude that part of CAIX present in plasma may originate from other organs made hypoxic upregulation as a consequence of preeclampsia. CAIX is generally expressed in very few normal tissues, but it is considered a specific marker of tissue hypoxia (Swietach et al., 2010). It is increasingly being studied as an important mediator of cancer cell response to hypoxic microenvironments. The extravillous trophoblast cells show phenotypic similarities to tumor cells with the same capacity for proliferation, migration and invasiveness, angiogenesis, and immune tolerance by exploiting analogous molecular mechanisms (Louwen et al., 2012). Moreover, the acidic microenvironment induces upregulation of both the expression and activity of CAIX in cancer cells and their exosomes, together with increasing the number of released exosomes (Logozzi et al., 2019). Similarly, women who will develop PE have a higher number of exosomes in the maternal circulation (Burkova et al., 2021). The oxygen concentration plays a crucial role in the regulation of biogenesis and secretion of placental exosomes. Exosomes release from trophoblast rise under low oxygen tension (Burkova et al., 2021). The main limitations to the study are related to the relative small sample size of the subjects enrolled; however, this is well offset by the meticulousness of the data collection and pregnancy longitudinal follow-up. Our results should be validated in a larger cohort of pregnant women at risk for the disease in order to confirm the clinical usefulness. Our assay based on enzyme-linked immunosorbent assay (ELISA) could be easily validated in further follow-up studies in different clinical laboratories. In conclusion, the identification of women at an increased risk of developing PE based on CAIX analysis seems to be possible starting from the 28th gestational week. However, we were unable to establish whether CAIX could represent an effective predictive biomarker of PE beyond 32 weeks of gestation because we did not have useful data after this gestational week. An earlier prediction of PE is useful in allowing the use of aspirin, the only preventative treatment that is proven to be effective, before the 16th week of gestation. However, in women at the high risk to develop PE as our cohort, a preventative treatment with aspirin was already implemented (Rolnik et al., 2017). Therefore, in women at a risk of developing PE, circulating concentrations of CAIX along with close maternal and fetal surveillance appear to be a suitable predictive biomarker for assessing short-term progression to preeclampsia.

## Data Availability

The raw data supporting the conclusion of this article will be made available by the authors, without undue reservation.
